# A decision tree for predicting the causative pathogens of community-acquired pneumonia from thin-section computed tomography

**DOI:** 10.1007/s11604-024-01691-4

**Published:** 2024-11-06

**Authors:** Haruka Sato, Fumito Okada, Yusuke Nakao, Yoshiki Asayama

**Affiliations:** 1 Department of Radiology, Faculty of Medicine, Oita University, 1-1 Idaigaoka, Hasama-Machi, Yufu, Oita 879-5593 Japan; 2 Department of Radiology, Oita Prefectural Hospital, Oita, Japan

**Keywords:** Chest, Community-acquired pneumonia, Computed tomography, Decision tree

## Abstract

**Purpose:**

To determine whether decision trees are useful for predicting organisms that cause community-acquired pneumonia (CAP).

**Materials and methods:**

We developed a decision tree for predicting the organisms that cause CAP based on previously reported characteristic computed tomography findings. Sixteen readers (two student doctors, six residents, and eight radiologists) separately diagnosed 68 randomly selected cases of CAP using chest computed tomography. The first, second, and third most likely causative organisms were estimated for each case, and the percentages of correct answers were evaluated for consistency with the isolated organisms. The same 68 cases were then read again using the decision tree, with the first three most likely organisms again being estimated, and the percentage of agreement was evaluated as the percentage of correct responses after using the decision tree.

**Results:**

For student doctors, residents, and radiologists, the percentage of correct responses increased significantly (*p* < 0.0001) when the decision tree was used to predict the first, second, and third most probable causative organism. The radiologists all obtained an accuracy rate of 80% or higher when estimating up to three candidate organisms using the decision tree. The organism for which the decision tree was most useful was *Mycoplasma pneumoniae*, followed by *Haemophilus influenzae* and *Chlamydophila pneumoniae* (*p* < 0.001).

**Conclusion:**

Use of the decision tree made it possible to estimate the organisms responsible for CAP with a high correct response rate.

## Introduction

Pneumonia is a common respiratory disease caused by pathogens such as *Streptococcus pneumoniae* and *Mycoplasma pneumoniae*, and is an important cause of death [1]. Mortality associated with pneumonia is influenced by initial antibiotic treatment. Traditionally, empirical antibiotic therapy is given first, and subsequent antibiotic therapy is adjusted according to the results of susceptibility testing. Bacterial culture results are available in 3–5 days, but their culture positivity rate is only 40% [2]. It is important to identify the risk factors associated with each causative pathogen and to evaluate radiological findings so that no time is lost in initiating appropriate management.

Although several studies have presented clinical and microbiological findings in patients with community-acquired pneumonia (CAP), there is a lack of characteristic clinical information from which to infer causative pathogens. In recent years, several reports have been published describing the thin-section computed tomography (CT) findings of pneumonia caused by a variety of microorganisms. The imaging features of pulmonary infections are closely related to the pathogenesis of pneumonia [3], with thin-section CT scans enabling clear identification of secondary pulmonary lobules and alveolar and bronchiolar involvement of the infection. Pneumonia can be divided into two patterns according to the different pathological and radiological characteristics of the causative pathogens: lobar pneumonia and bronchopneumonia [4, 5]. Lobar pneumonia exudes edematous fluid and neutrophils fill the alveolar space, resulting in CT findings of a non-segmental distribution. In contrast, a histological feature of bronchopneumonia is that inflammation is confined to the periphery of the bronchioles, resulting in a segmental distribution of CT findings. In addition, the frequencies of CT findings, such as bronchial wall thickening and centrilobular nodules, may differ between each pneumonia pattern (Table 1) [6–14].

**Table 1:** Frequencies of CT findings in each type of pneumonia

Causative pathogen	References	Age (mean) (y)	CT Findings			
			Centrilobular nodules	Bronchial wall thickening	Abscess	Cavitary nodule
Air space pneumonia						
*Streptococcus pneumoniae*	Okada F et al. [6]	22–98 (61.3)	17/86 (19.8)	22/86 (25.6)	0/86 (0)	0/86 (0)
*Chlamydophila pneumoniae*	Okada F et al. [7]	20–82 (57.7)	3/40 (7.5)	14/40 (35.0)	0/40 (0)	N/A
*Klebsiella pneumoniae*	Okada F et al. [8]	18–97 (61.5)	8/198 (4.0)	52/198 (26.3)	1/198 (0.5)	0/198 (0)
*Legionella pneumophilia*	Ito A et al. [9]	58–82 (67.1)	N/A	N/A	0/11 (0)	0/11 (0)
Bronchopneumonia						
*Mycoplasma pneumoniae*	Okada F et al. [7]	15–78 (47.3)	38/42 (90.5)	37/42 (88.1)	0/42 (0)	
*Haemophilus influenzae*	Okada F et al. [10]	16–91 (63.9)	137/211 (64.9)	181/211 (85.8)	0/211 (0)	0/211 (0)
*Staphylococcus aureus*	Morikawa K et al. [11]	24–98 (79.0)	53/68 (63.9)	63/68 (75.9)	3/83 (3.6)	N/A
*Moraxella catarrhalis*	Okada F et al. [12]	28–102 (74.9)	79/109 (72.5)	85/109 (78.0)	0/109 (0)	N/A
*Streptococcus milleri*	Okada F et al. (13)	20–88 (63.1)	9/33 (27.3)	23/33 (70.0)	7/33 (21.2)	0/33 (0)
*Nocardia*	Sato H et al. [14]	39–83 (67.9)	0/18 (0)	1/18 (5.6)	N/A	12/18 (66.7)

Data in parentheses are percentage

*N/A* not applicable

No previous attempts to use imaging findings to estimate the microorganisms responsible for CAP have been reported. In this study, we attempted to create a decision tree to determine the causative pathogen in adult patients with CAP, based on the frequencies of causative pathogens, clinical findings such as the peak age of onset, and the characteristic CT findings in each type of pneumonia. The usefulness of the decision tree for estimating the organisms that cause CAP was examined.

## Materials and methods

Our institutional review board approved this retrospective study (5–226) and waived the requirement for informed consent because of its retrospective nature.

### Make-up of decision tree

**Fig. 1:**
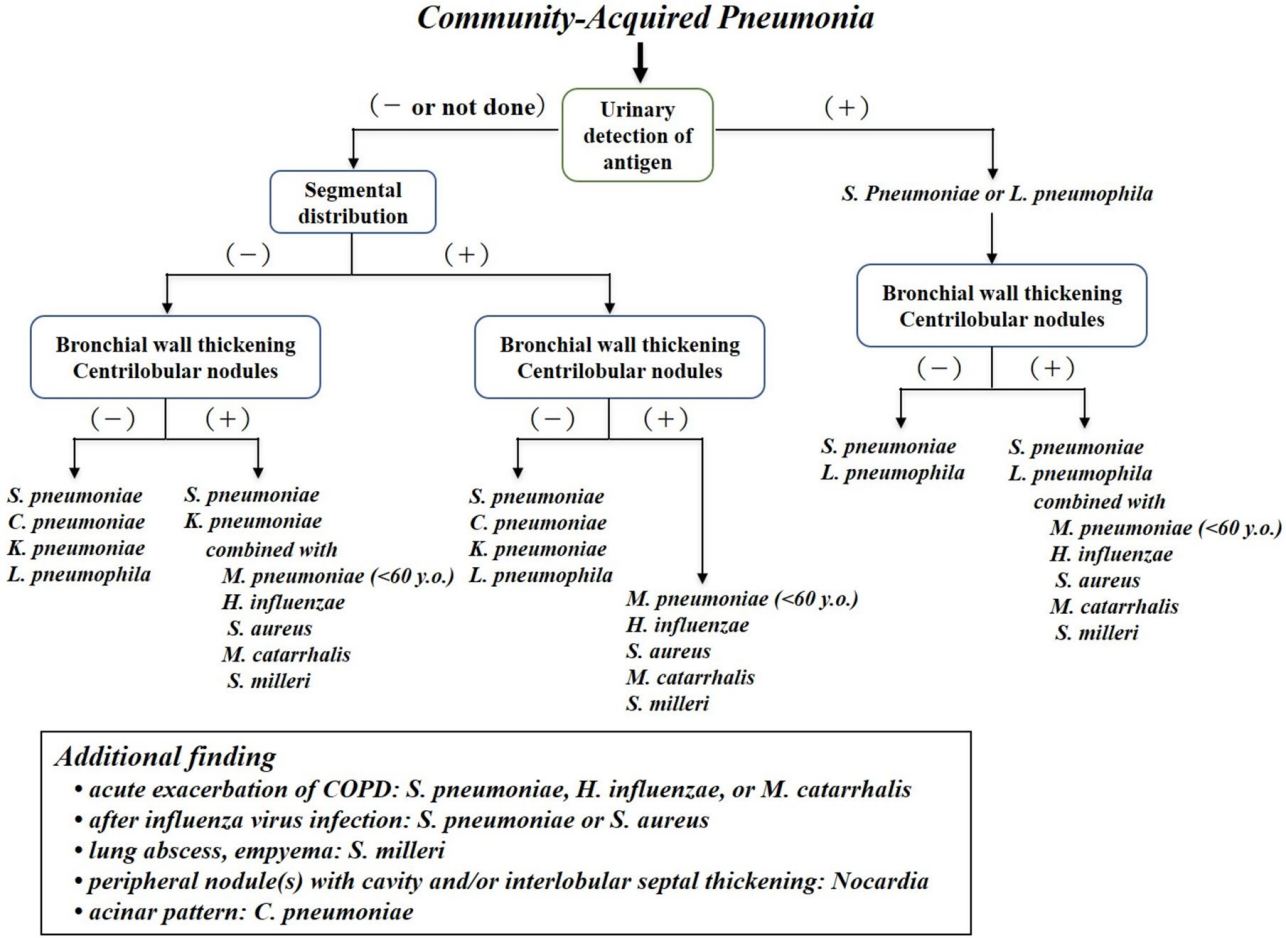
The decision tree for estimating the causative organisms of community-acquired pneumonia

Based on the CT findings of 929 previously reported CAP cases caused by one or more of the top 10 causative pathogens from January 1991 to June 2016, frequencies of CT findings of pneumonia caused by each pathogen were calculated (Table 1), and a decision tree was created to estimate causative pathogens referring to Table 1 (Fig. 1) [6–15]. Cases included single (*n* = 816) and concurrent infections (*n* = 113) caused by previously reported causative pathogens, including *S. pneumoniae* (*n* = 86), *Chlamydophila pneumoniae* (*n* = 40), *Klebsiella pneumoniae* (*n* = 198), *Legionella pneumophila* (*n* = 11), *M. pneumoniae* (*n* = 42), *Haemophilus influenzae* (*n* = 211), *Staphylococcus aureus* (*n* = 68), *Moraxella catarrhalis* (*n* = 109), *Streptococcus milleri* (*n* = 33), *Nocardia* (*n* = 18), *S. pneumoniae* combined with *H. influenzae* (*n* = 36), *S. pneumoniae* combined with *S. aureus* (*n* = 22), and *K. pneumoniae* combined with *S. aureus* (*n* = 55) (Fig. 1; Table 1) [6–15].

To determine whether pneumonia was caused by the microorganisms of bronchopneumonia or lobar pneumonia with reference to previously reported CT and pathological findings, a decision tree was first created by classifying abnormal CT findings as segmentally or non-segmentally distributed, then focusing on the presence of bronchial wall thickening and centrilobular nodules (Fig. 1). After inferring whether the pneumonia was caused by the microorganisms of bronchopneumonia or alveolar pneumonia, each microorganism was ordered from the highest to the lowest frequency based on the statistical frequencies of the causative pathogen (Fig. 1). Certain characteristic CT findings have been documented among the types of pneumonia caused by each of the organisms; the following features were included in the additional findings of the decision tree: an acinar pattern (a lobular unit consolidation or ground-glass opacity, characteristic of *C. pneumoniae*) [7, 16], pulmonary nodules with both cavity and/or interlobular septal thickening that are characteristic of *Nocardia* [14], and consolidation with a cavity and/or air suggestive of lung abscess and pleural effusion with thickened parietal and visceral pleural layers (i.e., split pleural sign) suggestive of empyema that are characteristic of *S. milleri* [13]. We also considered the patient’s age at onset, based on previous reports and the Japanese Respiratory Society guidelines for the management of pneumonia in adults [17–22]. Additional findings added to the decision tree were that the frequencies of infections with *S. pneumoniae*, *H. influenzae*, and *M. catarrhalis* are higher in patients with emphysema [12, 23, 24], and that both *S. pneumoniae* and *S. aureus* infections are more common after influenza virus infections [25, 26]; these were added as they have been reported as important clinical findings for estimation of causative pathogens.

### Selection of subjects

The 68 cases of CAP caused by the top ten causative organisms in our hospitals were randomly selected (Table 2). Diagnosis was established by isolation of the pathogen(s), serological tests, clinical features, and pulmonary infiltrates on chest radiographs. A patient was considered to have CAP if they presented with cough, fever, leukocytosis, or leukopenia at the time of hospital admission. None of the patients had been admitted to or treated in a hospital 2 weeks before admission. The populations of the causative pathogens in this image interpretation experiment were similar to those seen in Japan and the rest of the world [17–22].

**Table 2:** Pathogens responsible for pneumonia in the image interpretation experiment

Causative pathogens of pneumonia	Number of cases (*n* = 68)
*Streptococcus pneumoniae*	15 (22.1)
*Mycoplasma pneumoniae*	14 (20.6)
*Haemophilus influenzae*	9 (13.2)
*Chlamydophila pneumoniae*	7 (10.3)
*Staphylococcus aureus*	4 (5.9)
*Moraxella catarrhalis*	4 (5.9)
*Klebsiella pneumoniae*	4 (5.9)
*Streptococcus milleri*	3 (4.4)
*Nocardia*	2 (2.9)
*Legionella pneumophila*	1 (1.5)
*Concurrent infections*	
*S. pneumoniae* + *Mycoplasma pneumoniae*	4 (5.9)
*S. pneumoniae* + *Moraxella catarrhalis*	1 (1.5)

Data in parentheses are percentage

### CT interpretation

Two student doctors, six residents, and eight radiologists (excluding chest radiologists) independently assessed thin-section chest CT images of the 68 cases of CAP. CT examinations were performed with a variety of multi-detector CT scanners, with 1–1.25-mm reconstruction and coverage from the apex of the lung to the diaphragm. The scans were obtained with the patient in the supine position at full inspiration. Images were captured using window settings that allowed viewing of the lung parenchyma (window level − 600 HU; window width 1500 HU).

The first, second, and third candidates for the causative organisms were estimated, and the percentage of correct answers was evaluated based on whether the results were consistent with the actual organisms confirmed through laboratory testing. Readers were informed in advance that mixed infection cases were included; in the case of mixed infection, the correct answer was determined by matching all of the organisms involved. The same 68 cases were then read again using the decision tree to estimate up to three pathogen candidates, and the rate of agreement was evaluated as the percentage of correct responses after the decision tree was used. The readers were provided with only age and sex as patient information; clinical information such as the results of blood tests, urinary antigens, clinical manifestations, and underlying diseases were not provided.

### Statistical analysis

The primary endpoint was the percentage of correct responses for the first candidate for the causative agent, and the secondary endpoints were the percentage of correct responses where the causative agent was included in the second or third candidates. The significance of the mean of the correct response rate for each endpoint was examined using a *t* test. For each of the primary endpoints, a chi-square test was used to test for differences in the percentage of correct responses before and after using the decision tree, with a significance level of *p* = 0.05.

## Results

**Fig. 2:**
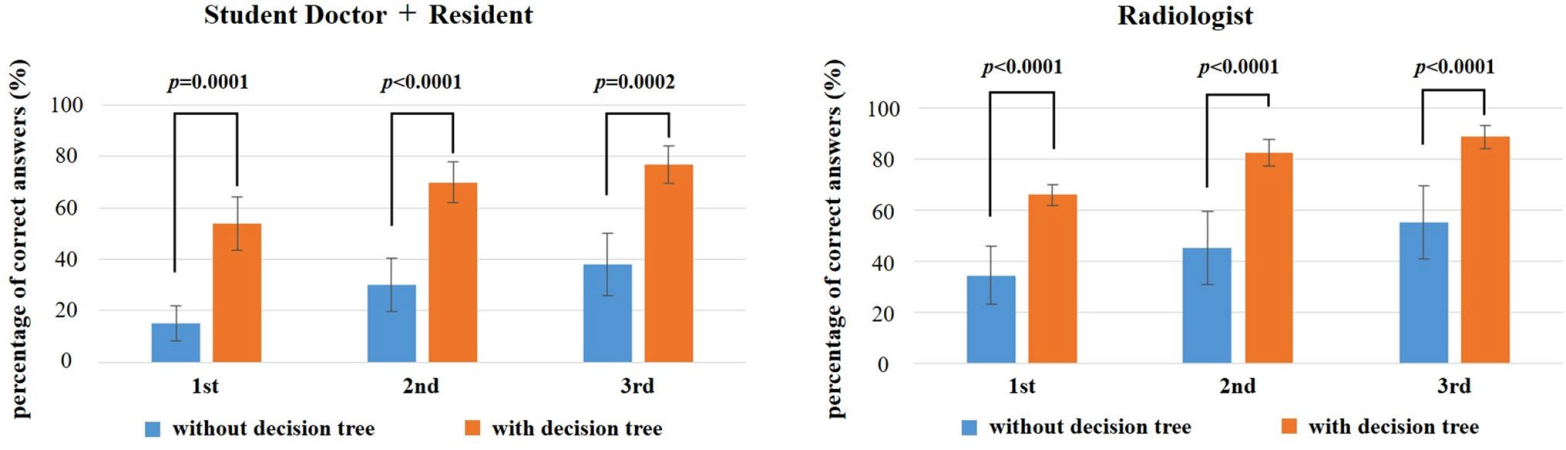
Percentage of correct answers among the first, second, and third candidates for student doctors, residents, and radiologists

**Fig. 3:**
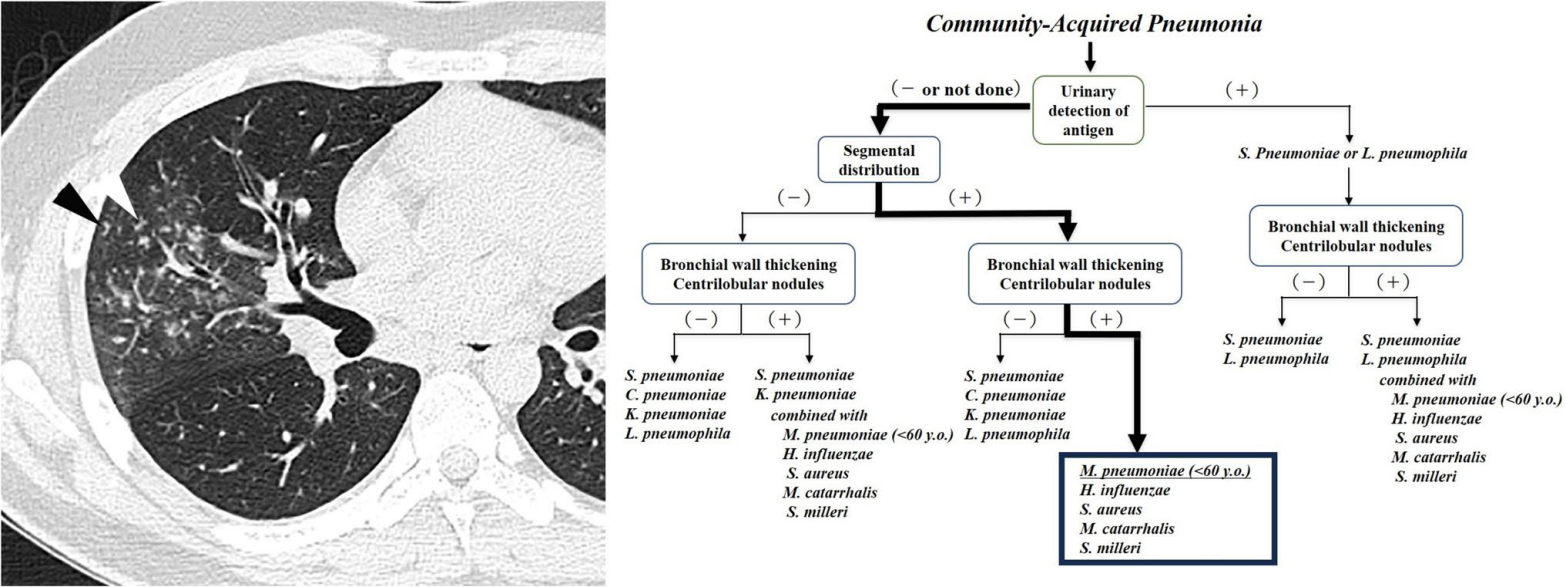
**a** and **b** *Mycoplasma pneumoniae* pneumonia in a 21-year-old man (Case 4). Transverse thin-section computed tomography (CT) at the level of the division of the middle lobe bronchus showed ground-glass opacity with a segmental distribution, bronchial wall thickening, and centrilobular nodules (arrows). These findings strongly suggest *M. pneumoniae* as the causative pathogen, because the pathogen is likely to be one that causes bronchopneumonia and the patient is under 60 years of age. The correct response rates for the first candidate for student doctors + residents, as well as radiologists, were 0% and 37.5%, respectively, before using the decision tree and 100% and 87.5% after using the decision tree

**Fig. 4:**
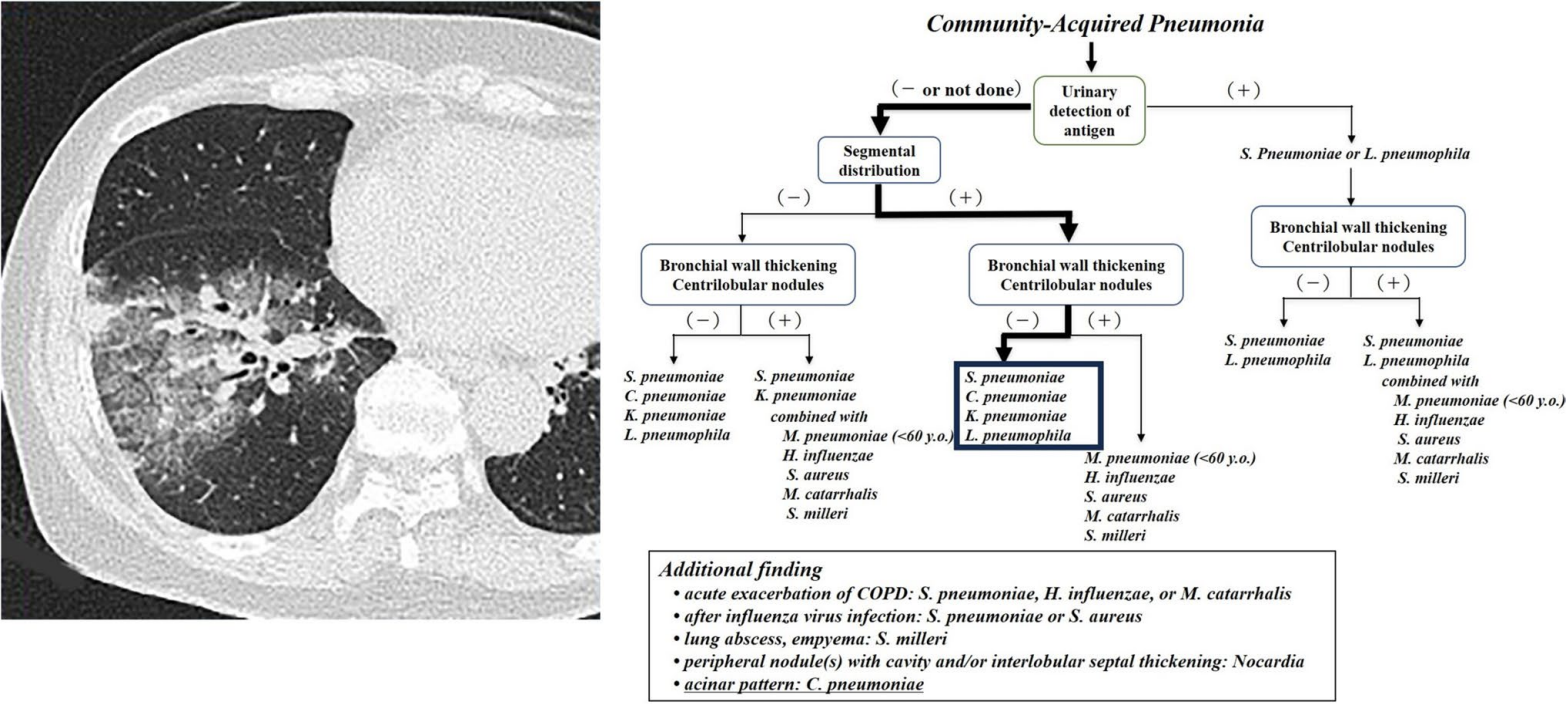
**a** and **b** *Chlamydophila pneumoniae* pneumonia in a 72-year-old woman (Case 18). Transverse thin-section CT at the level of the right lower lobe showed ground-glass opacity and consolidation with a segmental distribution. Bronchial wall thickening or centrilobular nodules could not be seen. These findings suggest that the causative pathogen was likely to be a pathogen causing lobar pneumonia. Additionally, because an acinar pattern can be observed, *C. pneumoniae* is suggested. The correct response rates for the first candidate for the student doctors + residents, as well as radiologists, were both 0% before using the decision tree, and both 62.5% after using the decision tree

**Fig. 5:**
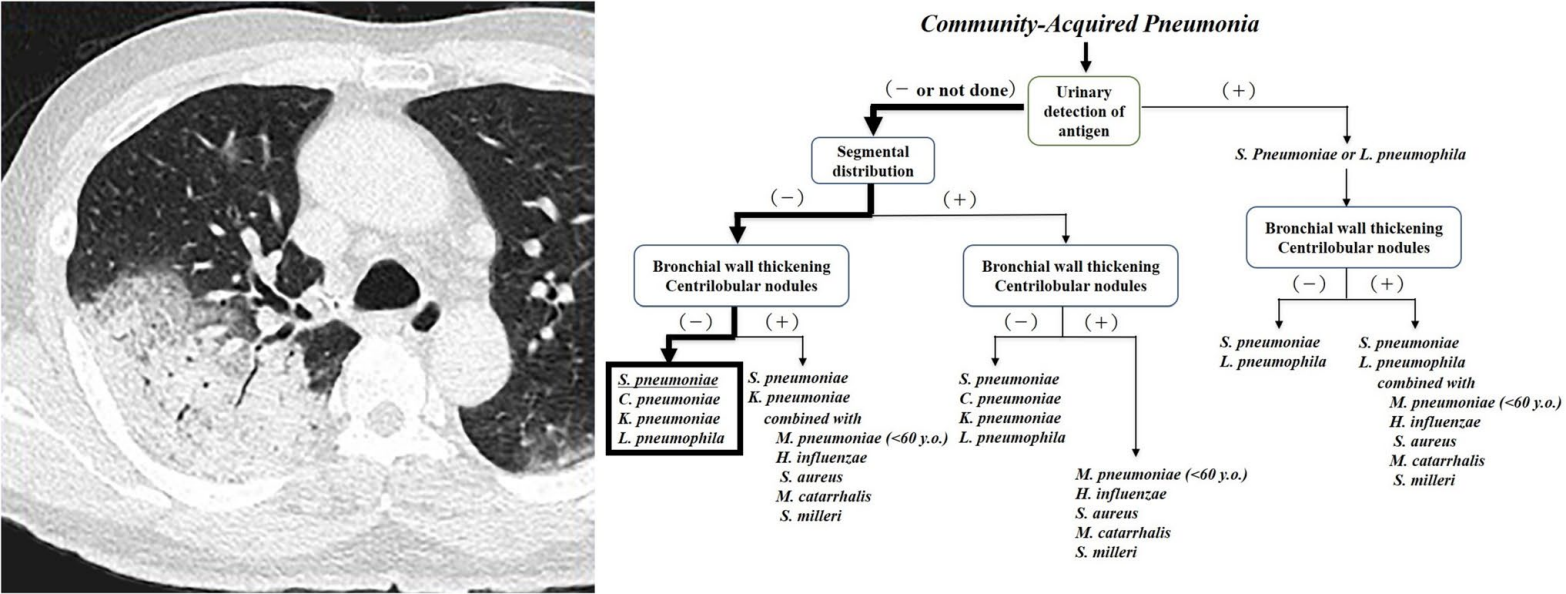
**a** and **b** *Streptococcus pneumoniae* pneumonia in a 66-year-old woman (Case 53). Transverse thin-section CT at the level of the division of the right B^10^ showed consolidation and ground-glass opacity with a non-segmental distribution. Bronchial wall thickening or centrilobular nodules cannot be seen. These findings suggest that the causative pathogen was likely to be one causing lobar pneumonia. According to its statistical frequency, *S. pneumoniae* was suggested as the pneumonia pathogen. If the distribution was diagnosed as segmental, *S. pneumoniae* could also be the first candidate because of the absence of evident bronchial wall thickening and centrilobular nodules. The correct response rates for the first candidate for student doctors + residents, as well as radiologists, were 12.5% and 87.5%, respectively, before using the decision tree, and 87.5% and 100% after using the decision tree

For all readers (students, residents, and radiologists), the percentage of correct responses increased significantly (*p* < 0.0001) with the use of the decision tree to help decide the first, second, and third candidates for the causative organism (Fig. 2). In the radiologist group, use of the decision tree to estimate up to the third candidate resulted in a correct response rate of over 80% for all participants (Figs. 3, 4, 5).

Examining results by pathogen, the percentages of correct answers for the first candidate are presented in Table 3a. The student doctors and residents found the decision tree to be most useful for predicting *M. pneumoniae* (without decision tree: 26.8%, with decision tree: 86.6%), followed by *H. influenzae* (without: 5.6%, with: 65.3%), *C. pneumoniae* (without: 7.1%, with: 44.6%), *S. pneumoniae* (without: 20.8%, with: 60.8%), *S. milleri* (without: 8.3%, with: 79.2%), *S. aureus* (without: 18.8%, with: 50.0%), and *S. pneumoniae* with *M. pneumoniae* (without: 0%, with: 9.4%) (*p* < 0.001). In contrast, no benefit was observed for *M. catarrhalis* (without: 9.4%, with: 0%), *K. pneumoniae* (without: 6.3%, with: 0%), *L. pneumophila* (without: 37.5%, with: 0%), or *S. pneumoniae* with *M. catarrhalis* (without: 0%, with: 0%).

**Table 3:** Percentage of correct answers

a. Percentage of correct answers for the first candidate						
Causative pathogen	Decision tree					
	Student doctor + Resident			Radiologist		
	Without	With	*p* value	Without	With	*p* value
*Mycoplasma pneumoniae*	30/112 (26.8)	97/112 (86.6)	< 0.001	59/112 (52.7)	107/112 (95.5)	< 0.001
*Haemophilus influenzae*	4/72 (5.6)	47/72 (65.3)	< 0.001	16/72 (22.2)	55/72 (76.4)	< 0.001
*Chlamydophila pneumoniae*	4/56 (7.1)	25/56 (44.6)	< 0.001	7/56 (12.5)	34/56 (60.7)	< 0.001
*Streptococcus pneumoniae*	25/120 (20.8)	73/120 (60.8)	< 0.001	78/120 (63.0)	83/120 (69.2)	N.S
*Streptococcus milleri*	2/24 (8.3)	19/24 (79.2)	< 0.001	5/24 (20.8)	23/24 (95.8)	< 0.001
*Staphylococcus aureus*	6/32 (18.8)	16/32 (50.0)	< 0.001	2/32 (6.3)	16/32 (50.0)	< 0.001
*Nocardia*	4/16 (25.0)	13/16 (81.3)	< 0.001	12/16 (75.0)	15/16 (93.8)	N.S
*Moraxella catarrhalis*	3/32 (9.4)	0/32 (0.0)	N.S	2/32 (6.3)	0/32 (0.0)	N.S
*Klebsiella pneumoniae*	2/32 (6.3)	0/32 (0.0)	N.S	1/32 (3.1)	2/32 (6.3)	N.S
*Legionella pneumophila*	3/8 (37.5)	0/8 (0.0)	N.S	1/8 (12.5)	0/8 (0.0)	N.S
*Streptococcus pneumoniae*						
+ *Mycoplasma pneumoniae*	0/32 (0.0)	3/32 (9.4)	< 0.001	5/32 (15.6)	20/32 (62.5)	< 0.001
+ *Moraxella catarrhalis*	0/32 (0.0)	0/32 (0.0)	-	0/32 (0.0)	0/32 (0.0)	-

b. Percentage of correct answers up to the third candidate						
Causative pathogen	Decision tree					
	Student doctor + Resident			Radiologist		
	Without	With	*p* value	Without	With	*p* value
*Mycoplasma pneumoniae*	44/112 (39.3)	103/112 (92.0)	< 0.001	75/112 (67.0)	109/112 (97.3)	< 0.001
*Haemophilus influenzae*	36/72 (50.0)	63/72 (87.5)	< 0.001	32/72 (44.4)	63/72 (87.5)	< 0.001
*Chlamydophila pneumoniae*	15/56 (26.8)	34/56 (60.7)	< 0.001	18/56 (32.1)	46/56 (82.1)	< 0.001
*Streptococcus pneumoniae*	65/120 (54.2)	100/120 (83.3)	< 0.001	94/120 (78.3)	103/120 (85.8)	0.025
*Streptococcus milleri*	6/24 (25.0)	19/24 (79.2)	< 0.001	8/24 (33.3)	24/24 (100)	< 0.001
*Staphylococcus aureus*	15/32 (46.8)	30/32 (93.8)	< 0.001	13/32 (40.6)	31/32 (96.9)	< 0.001
*Nocardia*	7/16 (43.5)	13/16 (81.3)	0.002	14/16 (87.5)	15/16 (93.8)	< 0.001
*Moraxella catarrhalis*	10/32 (31.3)	31/32 (96.9)	< 0.001	11/32 (34.4)	32/32 (100)	< 0.001
*Klebsiella pneumoniae*	5/32 (15.6)	20/32 (62.5)	< 0.001	17/32 (53.1)	27/32 (84.4)	< 0.001
*Legionella pneumophila*	4/8 (50.0)	0/8 (0.0)	N.S	4/8 (50.0)	0/8 (0.0)	N.S
*Streptococcus pneumoniae*						
+ *Mycoplasma pneumoniae*	0/32 (0.0)	4/32 (12.5)	< 0.001	5/32 (15.6)	25/32 (78.1)	< 0.001
+ *Moraxella catarrhalis*	0/8 (0.0)	2/8 (25.0)	< 0.001	2/8 (25.0)	4/8 (50.0)	N.S

Data in parentheses are percentages

*N.S.* not significant

The radiologists found the decision tree to be most useful for predicting *M. pneumoniae* (without: 52.7%, with: 95.5%), followed by *H. influenzae* (without: 22.2%, with: 76.4%), *C. pneumoniae* (without: 12.5%, with: 60.7%), *S. milleri* (without: 20.8%, with: 95.8%), and *S. aureus* (without: 6.3%, with: 50.0%) (*p* < 0.001). A benefit was also observed in mixed infections of *S. pneumoniae* with *M. pneumoniae* (without: 15.6%, with: 62.5%; *p* < 0.001). The decision tree was also of benefit for predicting *S. pneumoniae* (without: 63.0%, with: 69.2%), *Nocardia* (without: 75.0%, with: 93.8%), and *K. pneumoniae* (without: 3.1%, with: 6.3%); however, the differences were not statistically significant. In contrast, no benefit was observed for the prediction of *M. catarrhalis* (without: 6.3%, with: 0%) and *L. pneumophila* (without: 12.5%, with: 0%), although the decreases in the percentage of correct responses were not significant.

The percentages of correct answers up to the third candidate are presented in Table 3b. The student doctors and residents found the decision tree to be useful for all pathogens (*Nocardia*, *p* = 0.002; other pathogens, *p* < 0.001), except for *L. pneumophila*. The radiologists also found the decision tree to be useful for all pathogens (*S. pneumoniae*, *p* = 0.025; other pathogens, *p* < 0.001), except *L. pneumophila* and *S. pneumoniae* with *M. catarrhalis*.

## Discussion

To estimate the causative pathogens of CAP, we constructed a decision tree that focused on the characteristics of the top ten known causative pathogens, focusing mainly on the distribution of lesions, centrilobular nodules, and bronchial wall thickening (findings that have previously been reported on thin-section CT) [6–16].

Pneumonia can be dichotomized into lobar pneumonia and bronchopneumonia [4, 5]; the former has a non-segmental distribution caused by the rapid spread of large amounts of exudate through inter-alveolar passages such as the pores of Kohn, while in bronchopneumonia, lesions form mainly from inflammatory cell infiltrates with little exudate, resulting in a segmental distribution. The causative pathogens of lobar pneumonia are almost exclusively *S. pneumoniae*, *C. pneumoniae*, *K. pneumoniae*, and *L. pneumophila* [4, 5, 7–9], whereas other organisms are known to cause bronchopneumonia. The frequency of bronchial wall thickening ranges between 16.7 and 35% in lobar pneumonia caused by the above four pathogens, whereas this frequency ranges from 75.9 to 88.1% in bronchopneumonia caused by pathogens such as *H. influenzae*, *M. pneumoniae,* and *M. catarrhalis* [6–8, 10–12, 27]. The frequencies of centrilobular nodules are 4.0–19.8% in lobar pneumonia and 56.3–90.5% in bronchopneumonia [6–8, 10–12]. The CT findings of centrilobular nodules and bronchial wall thickening are significantly more frequent with the causative organisms of bronchopneumonia than with those causing lobar pneumonia (Table 2). The initial lesions of lobar pneumonia may present with a segmental distribution, as seen in bronchopneumonia. However, even in the case of segmental lesions, the presence of centrilobular nodules and bronchial wall thickening is less frequent in lobar pneumonia than in bronchopneumonia, which can help distinguish the causative organisms. Segmental lesions with fewer centrilobular nodules and bronchial wall thickening should raise suspicion for initial lesions of lobar pneumonia.

The following findings were also added to the decision tree: the CT finding of an acinar pattern is suggestive of *C. pneumoniae* pneumonia [7, 16], and *S. milleri* is the most common pathogen causing pulmonary abscess and empyema [13, 28, 29]. Clinical findings that may be useful for estimating the causative pathogens were also reflected in the decision tree. According to previous reports, patients with *M. pneumoniae* pneumonia are generally younger than patients with pneumonia caused by other pathogens [6–15]. Based on previous reports and the Japanese Respiratory Society guidelines, the statistical frequency of the causative organisms of CAP and the age of onset were also reflected in the decision tree [6–15, 17–22]. For each group of endpoints in the decision tree, the candidate pathogens were ordered by statistical frequency. The following facts were also added to the decision tree as additional findings; *S. pneumoniae*, *H. influenzae*, and *M. catarrhalis* are frequently detected in CAP in emphysema patients [12, 23, 24], and *S. pneumoniae* and *S. aureus* account for the majority of infections following influenza virus infection [25, 26].

The decision tree reflects that *S. pneumoniae* is the most common pathogen in concurrent pneumonia in CAP, with concurrent pneumonia accounting for 30–66.4% of all *S. pneumoniae* pneumonia cases [6, 30–32]. Additionally, in a study of 962 *K. pneumoniae* pneumonia cases, 79.4% were reported to be mixed infections with other pathogens such as *S. aureus* [8]. Most cases of pneumonia caused by mixed infections are mixes of pathogens causing lobar pneumonia and those causing bronchopneumonia; mixed infections between multiple pathogens causing lobar pneumonia or bronchopneumonia are rare [6, 8]. Okada et al. compared the pulmonary thin-section CT findings of patients with *S. pneumoniae* pneumonia with and without concurrent infection [6]. Centrilobular nodules and bronchial wall thickening were significantly more frequent in patients with pneumonia caused by concurrent infection than in those infected with *S. pneumoniae* alone. Additionally, in a previous report comparing thin-section CT findings between patients with *K. pneumoniae* pneumonia alone and those with concurrent infection, findings of centrilobular nodules, bronchial wall thickening, and cavity were significantly more frequent in patients with concurrent pneumonia [15]. In contrast, mixed infections are less common in *C. pneumoniae* pneumonia [7]. These findings on concurrent infections were reflected in the decision tree.

The usefulness of the decision tree for estimating the organisms responsible for CAP was examined using two student doctors, six residents, and eight radiologists. The decision tree increased the percentage of correct answers for all examiners, especially the radiologists; estimation of the first three most likely candidates using the decision tree resulted in more than 80% correct answers for everyone. When the usefulness of the decision tree was examined according to the causative organisms, it was most useful for *M. pneumoniae*, followed by *H. influenzae* and *C. pneumoniae*.

Europe and the United States recommend de-escalation therapy for the treatment of CAP, whereas Japan recommends escalation therapy. In Japan, *S. pneumoniae*, *H. influenzae*, *M. pneumoniae*, and *C. pneumoniae* are the main organisms that cause CAP. Because most *S. pneumoniae* in Japan are macrolide-resistant, unlike in Europe and the United States, macrolides are not used as the first-line treatment for *S. pneumoniae*; instead, penicillins combined with β-lactamase inhibitors are recommended. An exception is *M. pneumoniae* and *C. pneumoniae,* which are treated as atypical pneumonia because penicillins in combination with β-lactamase inhibitors are ineffective; for these organisms, macrolides are the first-line therapy. Using the decision tree, atypical pneumonia cases caused by *M. pneumoniae* and *C. pneumoniae* could be predicted with high accuracy (residents, student doctors, and radiologists were all *p* < 0.001). The decision tree developed here may allow for early suspicion of atypical pneumonia and its early treatment.

There were several limitations to our study. This was a retrospective study, and only the top ten CAP causative bacterial pathogens were included in the decision tree. Additionally, cases of viral pneumonia were excluded from the present study; however, the CT findings of viral pneumonia are described in many reports and are quite different from the findings of bacterial pneumonia in otherwise healthy individuals, meaning that radiologists should not have any difficulty in differentiating between them [33]. The number of cases studied was also small. Additionally, the decision tree was unable to provide any insight into the prediction of *L. pneumophila* in either group. When the microorganisms responsible for lobar pneumonia were ordered from the highest to the lowest statistical frequency of the causative pathogens, *L. pneumophila* was the least frequent, ranking fourth (Fig. 1). It can be reasonably assumed that readers would be unable to select *L. pneumophila* as a causative organism if estimating causative organisms up to the third candidate; consequently, they would be unable to provide the correct answer. However, it has recently become possible to detect antigens for all types (type 1–15) of *L. pneumophila* in urine, and high diagnostic accuracy for this organism could have been obtained if the results of the urinary antigens were reflected in the present decision tree. In Japan, CT scans are usually performed before the results of urine antigen tests (*S. pneumoniae* and *L. pneumophila*) or blood tests, or at least before the results of these tests are available. To reflect this clinical reality in this study, the reading experiments were carried out without the results of urinary antigen tests or clinical findings being known.

In conclusion, we developed a decision tree to estimate the causative pathogens for CAP based on thin-section CT findings, patient characteristics, the statistical frequency of causative pathogens, the age of onset, and the Japanese Respiratory Society guidelines. The results of validation show that the decision tree is useful for estimating the causative pathogens in CAP. The addition of further patient information to the decision tree, such as urinary antigen results and clinical manifestations, may allow for an even higher accuracy rate.
